# Synthesis, Crystal Structures, Genotoxicity, and Antifungal and Antibacterial Studies of Ni(II) and Cd(II) Pyrazole Amide Coordination Complexes

**DOI:** 10.3390/molecules29051186

**Published:** 2024-03-06

**Authors:** Amal El Mahdaoui, Smaail Radi, Youssef Draoui, Mohamed El Massaoudi, Sabir Ouahhoud, Abdeslam Asehraou, Nour Eddine Bentouhami, Ennouamane Saalaoui, Redouane Benabbes, Koen Robeyns, Yann Garcia

**Affiliations:** 1 LCAE, Department of Chemistry, Faculty of Sciences, University Mohammed I, Oujda 60000, Morocco; amalelmahdaoui2@gmail.com (A.E.M.); youssefx7draoui@gmail.com (Y.D.); elmassaoudio@gmail.com (M.E.M.); 2 Laboratory of Bioresource Biotechnology Ethnopharmacology and Health, Faculty of Sciences, University Mohammed I, Oujda 60000, Morocco; sabir.ouahhoud@gmail.com (S.O.); a.asehraou@ump.ac.ma (A.A.); noureddine.bentouhami@ump.ac.ma (N.E.B.); e.saalaoui@ump.ac.ma (E.S.); r.benabbes@ump.ac.ma (R.B.); 3 Faculty of Medicine and Pharmacy, University Sultan Moulay Slimane, Beni Mellal 23000, Morocco; 4 Institute of Condensed Matter and Nanosciences, Molecular Chemistry, Materials and Catalysis (IMCN/MOST), Université Catholique de Louvain, Place Louis Pasteur 1, 1348 Louvain-la-Neuve, Belgium; koen.robeyns@uclouvain.be

**Keywords:** pyrazole, amide, coordination complexes, antibacterial and antifungal activity, genotoxicity

## Abstract

In this study, we synthesized two coordination complexes based on pyrazole-based ligands, namely 1,5-dimethyl-*N*-phenyl-1H-pyrazole-3-carboxamide (**L_1_**) and 1,5-dimethyl-*N*-propyl-1H-pyrazole-3-carboxamide (**L_2_**), with the aim to investigate bio-inorganic properties. Their crystal structures revealed a mononuclear complex [Ni(**L_1_**)_2_](ClO_4_)_2_ (**C_1_**) and a dinuclear complex [Cd_2_(**L_2_**)_2_]Cl_4_ (**C_2_**). Very competitive antifungal and anti-Fusarium activities were found compared to the reference standard cycloheximide. Additionally, **L_1_** and **L_2_** present very weak genotoxicity in contrast to the observed increase in genotoxicity for the coordination complexes **C_1_** and **C_2_**.

## 1. Introduction

The growing problem of antibiotic resistance poses a significant threat to global health and food security. Bacteria are becoming increasingly resistant to commonly used antibiotics, forcing the use of less effective alternatives for treating common infections. This has led to the emergence of multi-drug-resistant pathogens, which the World Health Organization has identified as a major global health concern. Therefore, there is an immediate need to discover new types of medicines that can effectively fight these resistant strains [1,2].

In the field of pharmaceuticals, most compounds, including lead structures, are typically organic in nature. This preference for organic compounds may seem obvious, given that metals and their compounds are primarily associated with industrial and catalytic applications and often linked to toxicity concerns. It is however interesting to note that metal-based coordination complexes have played vital roles in the history of medicine. Examples include arsenic’s use in the first effective syphilis treatment (Salvarsan), mercury in the antiseptic mercurochrome and vaccine preservative thiomersal, or gold in treating rheumatoid arthritis (Auranofin) [3].

We recently turned our interest to pyrazole molecules due to their versatility and ability to engage in extensive interactions, making them particularly advantageous in the design and synthesis of metal–organic coordination compounds [4,5,6,7,8,9,10,11,12,13]. Such complexes could be proposed for various applications such as metal ion extraction [14,15,16], catalysis [17], adsorbents of heavy metals [18,19,20,21,22], and corrosion inhibitors [23,24]. Additionally, pyrazole compounds have attracted considerable interest due to their broad spectrum of biological properties. These versatile compounds have shown significant potential as antibacterial, antifungal, anticancer, antioxidant, anti-inflammatory, antidepressant, antipyretic, antiviral, anti-tubercular, and anti-HIV agents [6,10,11,12,24,25,26,27,28]. The diverse range of biological effects exhibited by pyrazole molecules has made them a subject of extensive research in medicinal and pharmaceutical studies [29,30].

Metal complexes incorporating an amide functional group have gained considerable attention too. Amides possess broad biological properties and are utilized as effective chelating agents. Notably, amide groups incorporated into heterocyclic bases such as pyridine and pyrazine have emerged as a rapidly expanding class of ligand scaffolds that efficiently bind metal ions [31].

**Scheme 1:**
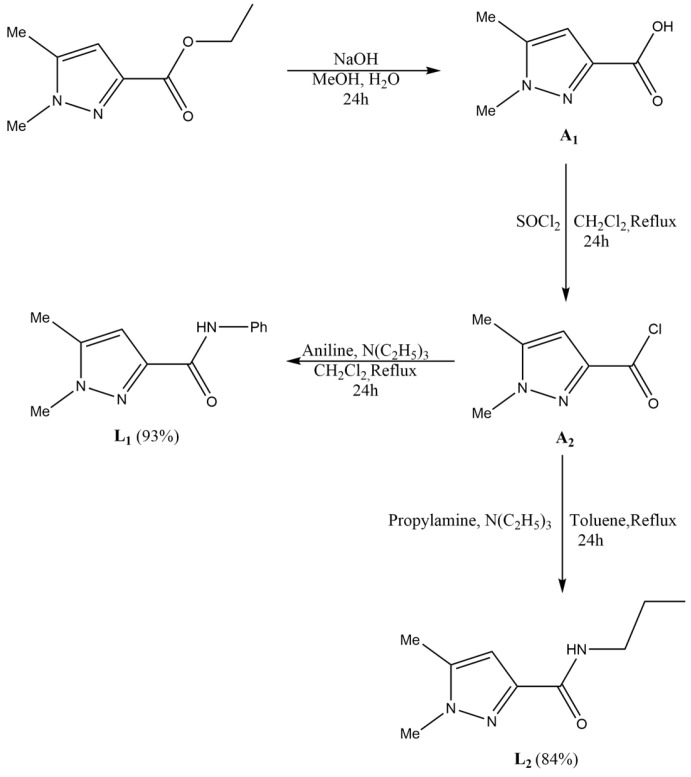
Synthetic pathways leading to the formation of the ligands **L_1_** and **L_2_**.

This study primarily focuses on the synthesis and characterization of two coordination complexes based on pyrazole amide ligands as antibiotic agents, namely 1,5-dimethyl-*N*-phenyl-1H-pyrazole-3-carboxamide (**L_1,_** Scheme 1) and 1,5-dimethyl-*N*-propyl-1H-pyrazole-3-carboxamide (**L_2_**). To do so, we examined the reactivity of **L_1_** in methanol with [Ni(H_2_O)_6_](ClO_4_)_2_ which led to a mononuclear complex [Ni(**L_1_**)_2_](ClO_4_)_2_ (**C_1_**). Additionally, we explored the reactivity in methanol of **L_2_** with CdCl_2_·2.5H_2_O, which led to a dinuclear complex [Cd_2_(**L_2_**)_2_]Cl_4_ (**C_2_**).

To assess the applicability of these compounds in real-world settings, their antibacterial and antifungal properties were studied. All compounds demonstrated antimicrobial activity against Gram (+) bacteria *Listeria innocua* and *Staphylococcus aureus*, as well as Gram (−) bacteria such as *Escherichia coli* and *Pseudomonas aeruginosa* and antifungal activity against pathogenic fungi *Geotrichum candidum*, *Aspergillus niger*, and *Penicillium crustosum.* Studying the genotoxicity of our compounds is also key to assessing their safety and evaluating their harmful effects for use cases in medicine, agriculture, and other applications. Minimal genotoxicity was observed for **L_1_** and **L_2_**, but a significant rise in genotoxicity was exhibited for **C_1_** and **C_2_**.

## 2. Results and Discussion

### 2.1. Synthesis of the Ligands

Since pyrazole has desirable biological properties, as outlined in the introduction, we used ethyl 1,5-dimethyl-1H-pyrazole-3-carboxylate as a starting point. Next, we performed the hydrolysis of the previous compound using methanol and NaOH to obtain 1,5-dimethyl-1H-pyrazole-3-carboxylic acid (**A_1_**). In the following step, **A_1_** was dissolved in dichloromethane along with thionyl chloride SOCl_2_ under stirring to obtain 1,5-dimethyl-1H-pyrazol-3-carbonyl chloride (**A_2_**). The goal of the previous hydrolysis and chlorination steps was to be able to add the amide function to our compound, which is known to have desirable biological properties as mentioned in the introduction. In addition, **A_2_** (Scheme 1) was combined with a mixture of triethylamine and aniline in CH_2_Cl_2_. The mixture was heated at reflux for 24 h, filtered, and concentrated. The final product (**L_1_**) was obtained as a white powder with a high yield (93%). Afterwards, **A_2_** was combined with a mixture of propylamine and triethylamine in toluene. The resulting mixture was refluxed for 24 h, filtered, and the solvent was removed. The crude product was purified via column chromatography on silica gel, affording **L_2_** as a white solid with a high yield (84%) (Scheme 1).

### 2.2. Synthesis of the Complexes

**Scheme 2:**
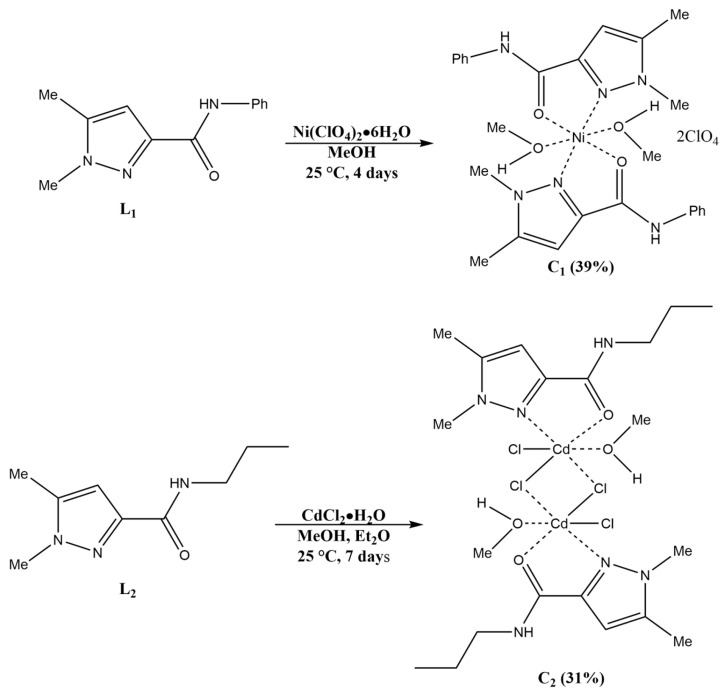
Synthesis of the coordination complexes **C_1_** and **C_2_**.

We aimed to investigate the influence of metal and counter anion selection on the formation and structural features of our coordination complexes, assessing their biological applications. To accomplish this, we utilized different ligand/metal ratios, experimented with diverse metal salts featuring varying counter anions, and employed various crystallization methods and solvents. From the compounds studied, **C_1_** and **C_2_** stood out as the most promising, producing high-quality single crystals suitable for X-ray analysis. The synthesis of the coordination complexes was conducted following Scheme 2. Here, a methanolic solution of Ni(ClO_4_)_2_·6H_2_O was added to a methanolic solution of **L_1_**. After four days of slow evaporation, blue single crystals of **C_1_** were obtained. A similar synthesis was carried out with **L**_2_ and CdCl_2_·H_2_O. Vapor diffusion with diethyl ether led to colorless single crystals of **C_2_**.

### 2.3. FT-IR Spectroscopy

**Figure 1:**
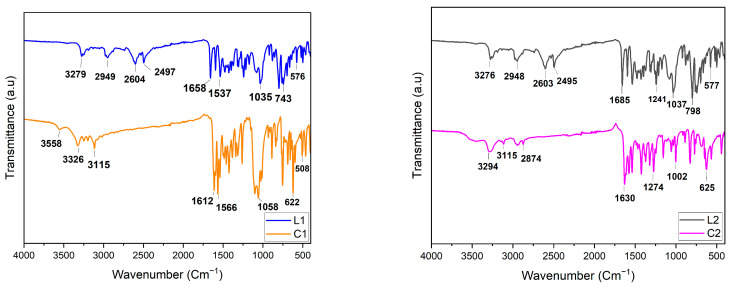
FTIR spectroscopy comparison plot of **L_1_**, **C_1_** (**left panel**) and **L_2_**, **C_2_** (**right panel**).

Figure 1 reveals FT-IR spectra of **L_1_**, **L_2_**, and their respective coordination complexes **C_1_** and **C_2_**. The purpose of this analysis was to observe the distinctive bands of the ligands and track their shifts upon coordination with the transition metals. Characteristic bands (2949(1) cm^−1^) of the aromatic C-H are observed in both **L_1_** and **L_2_**. Additionally, bands at 3279 cm^−1^ and 3276 cm^−1^ for **L_1_** and **L_2_**, respectively, reveal N-H vibrations of the amine groups. Additionally, amide C=O bands are observed at 1658 cm^−1^ and 1685 cm^−1^ for **L_1_** and **L_2_**, respectively. Furthermore, ~1537 cm^−1^ corresponds to C=C aromatic stretching in both **L_1_** and **L_2_**. C-O vibrations of the alkyl group function in **L_2_** are detected at 1037 cm^−1^ and 1241 cm^−1^.

By comparing **L_1_**, **C_1_** and **L_2_**, **C_2_**, respectively, a shift in the bands is systematically observed, which points to metal coordination. Furthermore, the peak at 1058 cm^−1^ becomes stronger in **C_1_** compared to **L_1_**, which can be explained by the presence of non-coordinating perchlorate anions [32]. Using FT-IR, we were unable to observe bands corresponding to Cd-O, Cd-N, Ni-O, and Ni-N, which are theoretically expected below 500 cm^−1^ [33,34,35]. However, we were able to confirm the formation of the respective complexes by X-ray crystallography.

### 2.4. X-ray Crystallography

**Figure 2:**
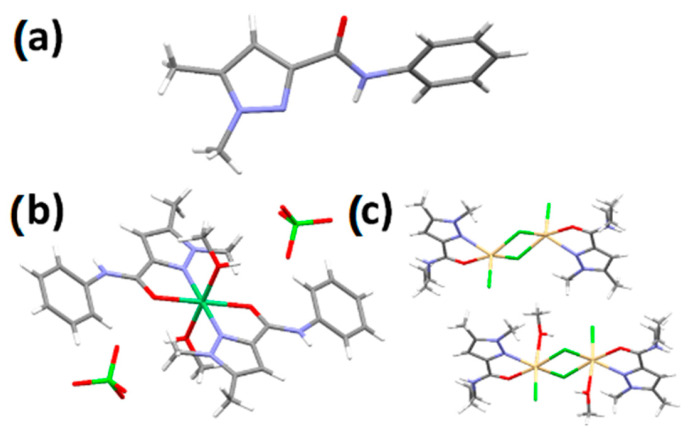
View of the molecular structures of **L_1_** (**a**), [Ni(**L_1_**)_2_](ClO_4_)_2_ (**C_1_**), (**b**) and [Cd_2_(**L_2_**)_2_]Cl_4_ (**C_2_**) (**c**). Disorder was omitted for clarity.

**L_1_** crystallizes in the orthorhombic system, in space group *Pbca* (#61). The asymmetric unit contains one ligand **L_1_** (*Z* = 8). A single hydrogen bond between the N-H and C=O propagates along the *b*-axis (Figure 2a). Complex **C_1_** crystallizes in the monoclinic system, in space group *P*2_1_/*n* (#14). The crystal structure shows a nickel mononuclear complex, the metal ion being coordinated by two bidentate chelating **L_1_** ligands in the equatorial position and two methanol molecules in axial positions (Figure 2b). The complex is perfectly symmetrical, with the Ni atom found at a crystallographic inversion center; the asymmetric unit consists thus of half a **C_1_** nickel complex with a single perchlorate as a counter anion (Z′ = 0.5).

Complex **C_2_** crystallizes in the orthorhombic system, in space group *Pca*2_1_ (#29). The asymmetric unit consists of two dinuclear complexes, one being solvated with two methanol molecules, giving an octahedral geometry around the Cd atoms, the other without any coordinated solvent molecules, giving a square pyramidal geometry for the Cd atoms. In either case, the Cd centers are complexed with a bidentate chelating **L_2_** ligand, one chloride anion, and two bridging chloride anions with a 3.74 Å Cd···Cd separation distance for the desolvated complex and a 3.60 Å Cd···Cd separation dinuclear for the solvated dinuclear complex.

### 2.5. Antibacterial Activity

**Figure 3:**
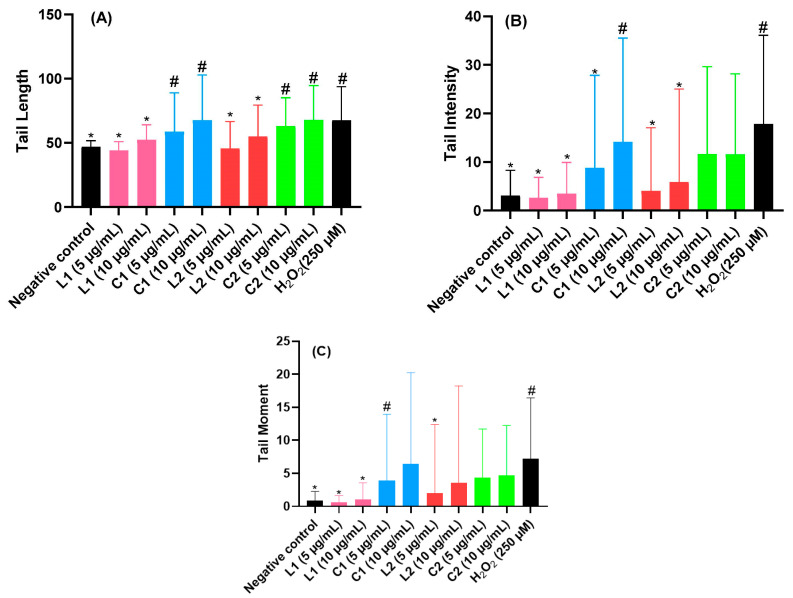
Assessment of the impact of **L_1_**, **L_2_** and **C_1_**, **C_2_** on (**A**) DNA tail length, (**B**) the percentage of tail intensity, and (**C**) DNA tail moment in rat leukocytes. Results presented as means ± SEM (50 cells × 2). # *p* < 0.05 compared with negative control; * *p* < 0.05 compared with H_2_O_2_.

Screening of the antibacterial activities of **L_1_**, **L_2_**, **C_1_**, and **C_2_** against Gram (+) (*Listeria innocua* and *Staphylococcus aureus*) and Gram (−) (*Escherichia coli* and *Pseudomonas aeruginosa*) bacteria was undertaken. Interestingly, there is an increase in the inhibition of **C_1_** and **C_2_** compared to **L_1_** and **L_2_**_,_ respectively, despite the lower concentration of the coordination complexes (see Figure 3 and Table 1). This enhanced performance can be attributed primarily to the influence of transition metals, which increase the lipophilicity of the coordination complexes compared to organic molecules [36]. As a result, the lipid membrane bilayer becomes more permeable to these complexes, facilitating their transfer [36]. This effect is more pronounced for **C_2_** compared to **L_2_**. **C_2_** provides the highest inhibition compared to **L_1_**, **L_2_** and **C_1_** on all bacteria tested. **C_2_** displays a 16.1 mm inhibition zone on *E. coli*, which is half of the inhibition zone provided by the control, Gentamicin. It is therefore higher than the dinuclear complex [Cd_2_(dmamp)_2_(NCS)_2_]_n_ (where dmamp = 2-[(2-dimethylaminoethylimino)methyl]phenol) [37] and of the same range as another dinuclear complex, CdL_2_Cl_2_, where L = 2-(3,5,5′-trimethyl-1′H-[1,3′-bipyrazol]-1′-yl)acetonitrile [38] (see Table 1). This result could be attributed to the superior antibacterial activity of pyrazole. Finally, note that Cd and Ni salts used in the synthesis of our coordination complexes display a weak antibacterial activity [38] that does not significantly affect the activity of our coordination complexes (see Table 1).

### 2.6. Antifungal Activity

We now focus our attention on the antifungal activity of our ligands and complexes against *Geotrichum candidum, Aspergillus niger*, and *Penicillium crustosum.* Coordination complexes **C_1_** and **C_2_** exhibit a noticeable increase in the inhibition zone compared to both **L_1_** and **L_2_** (Table 2), similar to the observed behavior in the previous section for the antibacterial activity. Additionally, the antifungal activity of **C_1_** and **C_2_** is generally comparable to that of cycloheximide. In particular, the inhibition zone of **C_2_** against *A. niger* (24.5 ± 0.71 mm) and *P. crustosum* (26 ± 2.83 mm) is slightly higher, with statistical significance (*p* < 0.05), compared to the reference cycloheximide [40]. This result confirms that **C_2_** has a very strong antifungal attitude. Additionally, **C_2_** also has comparable antifungal activity compared to a Ni coordination complex [41] and a superior activity compared to other Cd and Ni coordination complexes [38,42,43,44] (see Table 2). Similar to the antibacterial activities, note that Cd and Ni salts only show a weak antifungal activity [38] (see Table 2).

### 2.7. Antifungal Activity on Mycelial Growth of Fusarium oxysporum f. sp. Albinidis

Our ligands and coordination complexes display anti-Fusarium properties in contrast to cycloheximide (see Table 3), but our coordination complexes show stronger anti-Fusarium properties compared to the ligands even though our complexes were used in lower concentrations. In particular, **C_2_** exhibits a remarkable inhibition of 96% even at low concentrations (78.9 µmol/L) compared to all other compounds, including cycloheximide. Its characteristics overcome reported values for ligand molecules [46,47] in addition to other Cd and Ni coordination complexes (Table 3). The anti-Fusarium inhibition activity of our **C_2_** is also comparable to the remarkable inhibition properties of two dinuclear Co and Cu complexes reported recently [38,44]. Remarkably, the Cd and Ni salts used for the synthesis of our coordination complexes have a weak anti-Fusarium activity [38] (Table 3).

### 2.8. Genotoxicity

To assess the genotoxicity of **L_1_** and **L_2_** as well as of the coordination complexes **C_1_** and **C_2_**, we report the tail intensity, tail length, and tail moment (see Figure 3). For the sake of comparison, we compare their genotoxicity with hydrogen peroxide, which is known for being a typical DNA-damage-inducing agent [47]. **L_1_** and **L_2_** have weak genotoxicity compared to the negative control, whereas an increase in genotoxicity is found for **C_1_** and **C_2_** in comparison to hydrogen peroxide. This effect is likely due to the fact that Ni and Cd have a relatively high genotoxic activity [48,49]. Note that Cd is a toxic metal that accumulates in tissues and can cause genotoxic effects, including DNA damage and chromosomal aberrations, and disrupt DNA repair mechanisms. Additionally, Cd inhibits DNA methylation, altering gene expression patterns. Chronic exposure to cadmium is associated with an increased cancer risk [50]. Nickel, widely used in industries like stainless steel production, is classified as a human carcinogen. It induces genotoxic effects through DNA damage, chromosomal aberrations, and interference with DNA repair mechanisms, particularly in occupations such as mining and welding [51].

## 3. Experimental Section

### 3.1. Materials and Instrumentation

Commercially available analytical-grade solvents and chemicals were used without any additional purification steps. ^1^H and ^13^C NMR spectra were acquired using a Bruker AC 300 spectrometer (Bruker, Billerica, MA, USA). ESI ionization was employed to acquire HRMS data using a Q Exactive ion trap spectrometer from Thermo Fisher Scientific (Waltham, MA, USA), enabling high-resolution mass spectrometry measurements for **C**_1_ and **C**_2_. For the compounds **L**_1_ and **L**_2_, we used a low-resolution (LXQ) ESI MS. FT-IR spectra were recorded on a JASCO FT/IR-4700 spectrometer (JASCO, Lexington, KY, USA) using an ATR attachment. Melting points were measured using a Koffler bench. Elemental analyses were performed using a EuroEA Elemental Analyzer (HEKAtech GmbH, Wegberg, Germany).

### 3.2. Synthesis Section

#### 3.2.1. Synthesis of Ethyl 1,5-Dimethyl-1H-pyrazole-3-carboxylate

Ethyl 5-methyl-1H-pyrazole-3-carboxylate 10 g (65 mmol) and tBuOK 7.9g (65 mmol) were introduced into anhydrous diethyl ether (230 mL). The reaction mixture was then heated under reflux for 1 h, and subsequently cooled using ice. Next, a solution of methyl iodide was added dropwise, and the mixture was refluxed for 4 h, then filtered and concentrated to dryness. To the resulting oil, 25 mL of hexane was added, and the solution was placed in a refrigerator overnight. The product obtained after filtration was in the form of yellowish crystals. M.p: 45 °C. Yield: 77 % (7.5 g). ^1^H-NMR ($CDCl_{3}$) *δ* ppm: 1.40 (t, 3H, CH_2_-CH_3_), 2.33 (s, 3H, -CH_3_), 3.90 (s, 3H, N-CH_3_), 4.35 (q, 2H, CH_2_-CH_3_), (s, 1H, Pz-H). IR (KBr, v(cm^−1^)): 1690 (C=O).

#### 3.2.2. Synthesis of 1,5-Dimethyl-1H-pyrazole-3-carboxylic Acid (**A**_1_)

Ethyl 1,5-dimethyl-1H-pyrazole-3-carboxylate (2 g, 11.89 mmol) and methanol (20 mL) were added to a NaOH (0.95 g, 23 mmol) aqueous solution (30 mL). The resulting mixture was stirred for 24 h. After cooling to 0°C, it was neutralized using dilute HCl, leading to a white precipitate. The precipitate was separated by filtration, washed with water (15 mL), and dried in a desiccator. M.p: 190 °C. Yield: 90% (1.8 g). ^1^H-NMR (DMSO-d6) *δ* ppm: 2.23 (s, 3H, C-C), 3.75 (s, 3H, C- N), 6.44 (s, 1H, C=CH-C). ^13^C-NMR (DMSO-d6) *δ* ppm: 11.2 (C-C-N), 37.1 (C-N-N), 108.3 (C=CH-C), 140.7 (C-C=C), 142.1 (CH-C-C=O), 163.9 (C-C-OH).

#### 3.2.3. Synthesis of 1,5-Dimethyl-1H-pyrazol-3-carbonyl Chloride (**A**_2_)

**A_1_** (2 g, 14.27 mmol) was dissolved in dichloromethane, CH_2_Cl_2_ (40 mL). Under stirring, thionyl chloride SOCl_2_ (2 mL) was added, and the mixture was heated to 90 °C for 24 h. Afterwards, the mixture was cooled. When CH_2_Cl_2_ and SOCl_2_ had completely evaporated, the resulting white solid was dissolved in dichloromethane and distilled water. The solution was then neutralized with sodium bicarbonate until reaching a pH = 5. The mixture was decanted using a separating funnel, and the organic phase was subsequently extracted with dichloromethane (20 mL). It was then dried using sodium sulfate and evaporated to dryness. A white solid was obtained. M.p: 80 °C. Yield: 70% (1.7 g). ^1^H-NMR ($CDCl_{3}$) *δ* ppm: 2.16 (s, 3H, C-C), 3.84 (s, 3H, C-N), 6.59 (s, 1H, C=CH-C). ^13^C-NMR ($CDCl_{3}$) *δ* ppm: 11.3 (C-C-N), 37.5 (C-N-N), 110.0 (C=CH-C), 141.5 (C-C=C), 142 (CH-C-C=O), 162 (C-C-Cl).

#### 3.2.4. Synthesis of 1,5-Dimethyl-N-phenyl-1H-pyrazole-3-carboxamide (**L**_1_)

A solution of **A_2_** (1 g, 6.30 mmol) (20 mL) was mixed with triethylamine (0.87 mL) and aniline (0.56 mL) in dichloromethane (10 mL), which was added slowly. The resulting mixture was heated at reflux for 24 h, followed by filtration, and was then cooled to room temperature and concentrated to dryness. The expected product was precipitated in petroleum ether and was purified by recrystallization in petroleum ether, resulting in a white powder. M.p: 150 °C. Yield: 93% (0.97 g). FT-IR: 3279(w), 2949(w), 2604(w), 2497(w), 1658 (s), 1537(s), 1035(s), 743(m), 576(w). ^1^H-NMR ($CDCl_{3}$) *δ* ppm: 2.23 (s,3H, C-C), 3.74 (s, 3H, C-N), 6.55 (s, 1H, C=CH-C), 7.03 (t, 1H, CH=CH-CH), 7.27 (t, 1H, CH=CH-CH), 7.61 (d, 1H, C-CH=CH), 8.61 (s, 1H, NH-C). ^13^C-NMR ($CDCl_{3}$) *δ* ppm: 11.3 (C-C-N), 36.6 (C-N-N), 106.4 (C=CH-C), 119.5 (C-CH=CH), 123.8 (CH-CH=CH), 128.9 (CH=CH-CH), 138.1 (NH-C-CH), 140.7 (C-C=CH), 145.1 (N=C-CH), 159.9 (O=C-NH). MS (ESI) (Methanol): *m/z* = 216 [M+H] ^+^ (calc. 215.2), (see Figures S1, S2 and S5).

#### 3.2.5. Synthesis of 1,5-Dimethyl-N-propyl-1H-pyrazole-3-carboxamide (**L**_2_)

To a solution of **A_2_** (0.60 g, 3.78 mmol) in dry toluene (10 mL) at 0 °C, a mixture of propylamine (0.65 mL, 3.73 mmol) and triethylamine (0.52 mL, 3.75 mmol) in toluene (20 mL) was slowly added. The resulting mixture was heated at reflux for 24 h. The reaction mixture was then filtered, and the solvent was removed under reduced pressure. The crude product was purified via column chromatography on silica gel (EtOAc/Hexane: 5/5), resulting in a white solid. M.p 72 °C. Yield 84% (0.51 g). FT-IR: 3276(w), 2948(w), 2603(w), 2495(w), 1685(s), 1241(m), 1037(s), 798(s), 577(w). ^1^H-NMR (DMSO) δ ppm: 0.83 (t, 3H, C-C), 1.47 (m, 2H, C-C-C), 2.25 (s, 3H, C-C), 3,14 (m, 2H, NH-C-C), 3.75 (s, 3H, C-N), 6.37 (s, 1H, C=CH-C), 7.92 (t, 1H, C-NH-C). ^13^C-NMR (DMSO) δ ppm: 11.3 (C-C-N), 11.9 (C-C), 23.1 (C-C-C), 36.8 (C-N-N), 40.6 (NH-C-C), 105.8 (C=CH-C), 140.6 (C-C=CH), 145.5 (N=C-C=O), 162.0 (O=C-NH). MS (ESI) (Methanol): *m/z* = 182 [M+H] ^+^ (calc.181.2), (see Figures S3, S4 and S6).

#### 3.2.6. Synthesis of [Ni(L_1_)_2_](ClO_4_)_2_ (**C**_1_)

**L_1_** (43.1 mg, 0.2 mmol, 2 equiv.) was dissolved in methanol (3 mL). Ni(ClO_4_)_2_·6H_2_O (36,6 mg, 0.1 mmol, 1 equiv.) was dissolved in methanol (3 mL) and added to the previous solution of **L_1_**. The mixture was stirred for 10 min at room temperature. After 4 days of slow evaporation, blue single crystals were obtained. Yield: 39% (29 mg). FT-IR: 3558(w), 3326(w), 3115(w), 1612 (s), 1566(s), 1058(s), 622(s), 508(m). HRMS (ESI) (CH_3_OH): *m/z* = 244.07280 [C_24_H_26_O_2_N_6_^58^Ni], (see Figure S7).

#### 3.2.7. Synthesis of [Cd_2_(L_2_)_2_]Cl_4_ (**C**_2_)

**L_2_** (36.2 mg, 0.1 mmol, 3 equiv.) was dissolved in methanol (3 mL). CdCl_2_·H_2_O (24.2 mg, 0.1 mmol, 1 equiv.) was dissolved in methanol (3 mL), with the addition of five drops of water, and then added to the previous solution of $L_{2}$. The mixture was stirred for 10 min at room temperature. The resulting clear liquid mixture was subjected to vapor diffusion with diethyl ether (10 mL) at room temperature. After 7 days, colorless crystals were obtained. Yield: 31% (21 mg). FT-IR: 3294(w), 3115(w), 2874(w), 1630(s), 1274(s), 1002(m), 625(s). HRMS (ESI) (CH_3_OH): *m/z* = 234.07423 [C_18_H_30_O_2_N_6_^106^Cd], (see Figure S8).

### 3.3. X-ray Crystallographic Studies

Diffraction data for **L_1_**, **C_1_**, and **C_2_** were collected on an MAR345 Image plate detector using a monochromated (Montel optics) microfocus Mo-Kα radiation (*λ* = 0.71073 Å) source (Incoatec IµS). Data integration and reduction were performed using the CrysAlis^PRO^ V 1.171.37.35 crystallographic software package and absorption correction was used [52].

The structures were solved by dual-space direct methods (SHELXT) [53] and refined by full matrix least squares on F^2^ using SHELXL [54]. All non-hydrogen atoms were refined anisotropically and hydrogen atoms were placed at the calculated positions with isotropic temperature factors fixed at 1.2 times the U_eq_ of the parent atoms (1.5Ueq for methyl and OH hydrogens).

In **L_1_**, methyl hydrogens showed rotational disorder and were refined as an idealized disordered methyl group with two positions rotated by 60°. In **C_1_**_,_ the Ni complex showed whole-molecule disorder. The octahedral Ni complex consists of two **L_1_** ligands in the equatorial plane with two methanol molecules in the axial positions, giving a perfectly symmetrical complex, with the Ni atom found at an inversion center. The disorder consists of a 90° rotation of the ligands within the same equatorial plane with a refined ratio of about 84/16. Additionally, the perchlorate anion is also disordered over two discrete positions in a 90/10 ratio. In **C_2_**, both dinuclear complexes in the asymmetric unit show disorder of the Cd cations, and, as a result, so do the coordinated Cl anion and methanol. The asymmetric unit could be seen as a mixture of the solvated dinuclear complex and the desolvated structure. As the overall geometrical differences between both structures are relatively small, both can be interchanged. As no deterioration of the diffraction was observed, it is less likely that the observed structure is an averaged structure where solvent evaporation occurred at the surface of the crystal during the measurement at ambient conditions, as this would result in non-stoichiometric ratios of both dinuclear complexes. Also, one of the propyl chains in both dinuclear complexes showed disorder that could be refined in two discrete positions in a 90/10 ratio.

Details of the crystallographic and refinement details for the compounds are listed in Table 4. CCDC 2290595-2290597 contain the supplementary crystallographic data for this paper. These data can be obtained free of charge from The Cambridge Crystallographic Data Centre via www.ccdc.cam.ac.uk/structures.

## 4. Biological Activity

### 4.1. Microorganisms and Culture Media

The bacterial strains utilized in this work were two Gram-positive (*Staphylococcus aureus* (*S. aureus*) and *Listeria innocua* (*Li. innocua*)) and two Gram-negative bacteria (*Pseudomonas aeruginosa* (*Ps. aeruginosa*) and *Escherichia coli* (*E. coli*)). These bacterial strains are involved in foodborne illnesses. The fungal strains tested in this work were *Asperillus niger*, *Geotrichum candidum*, and *Penicillium crustosum.* These fungi are involved in postharvest diseases of fruits and vegetables, and they can be associated with the production of mycotoxins, which are dangerous for human and animal health. All these microorganisms are available in the Laboratory of Bioresources, Biotechnology, Ethnopharmacology and Health, Univ. Mohammed Premier of Oujda. The culture media used for their culture were Mueller–Hinton medium (BIOKAR, Allonne, France) for bacteria and Potato Dextrose Agar (PDA) (BIOKAR, Allonne, France) for fungi.

### 4.2. Measurement of Antibacterial and Antifungal Activities

The solid-medium diffusion method (Mueller–Hinton medium) was used to determine antibacterial activity against bacteria (*Listeria innocua*, *E. coli*, *Pseudomonas aeruginosa*, and *Staphylococcus aureus*), and *Potato Dextrose Agar medium* (BIOKAR, France) for antifungal activity against *Asperillus niger*, *Geotrichum candidum*, and *Penicillium crustosum*. The strains were diluted and adjusted to 0.5 McFarland, which corresponds to 10^6^ CFU/mL for bacteria and 10^6^ spores/mL for molds. Cultures were diluted with Mueller–Hinton broth for bacteria, and with sterile physiological water for molds. An amount of 0.1 mL of each diluted strain was inoculated by surface spreading of the solid culture medium corresponding to each type of microorganism (bacteria or mold). An amount of 100 μL of each sample at a concentration of 6 mg/mL (DMSO) was filled into the wells made on the solid medium. The dishes were then incubated for 2 h at 4 °C and then at 37 °C for 24 h for bacteria and at 25 °C for fungi. The diameters of the inhibition zones obtained around the wells were measured. Cycloheximide and Gentamicin were used as positive controls against fungi and bacteria, respectively.

### 4.3. Antifungal Activity on Mycelial Growth of Fusarium oxysporum

*Fusarium oxysporum* isolates were obtained from xylem tissues showing typical “Bayoud” symptoms on Boufegousgharas date palm in Figuig, Morocco. Small vascular tissue fragments were removed and placed in sterile PDA medium, then incubated at 28 °C. The identification of *Fusarium oxysporum* isolates was carried out by observing their morphological features [55,56]. Following incubation on PDA medium at 28 °C, a single monoconidial *Fusarium oxysporum* was isolated.

A total of 4 mg of each molecule was dissolved in DMSO (1 mL). From this solution, 50 µL and 150 µL were placed in sterile tubes. Then, the sterile liquid PDA was added until reaching a total volume of 10 mL. The entire set was placed on Petri dishes with 8.5 cm diameter, and the medium was allowed to stand until solidification [51]. In the middle of each Petri dish was a pellet of FOA that had already grown on the solid PDA. The dishes were incubated for four days at 28 °C. By comparing the diameter of the FOA to a control that contained only DMSO at some dose (control), the results are reported as a percentage of inhibition as follows [52]:


$$\%\;of\;inhibition=\frac{D_{0}-D_{x}}{D_{0}}\times 100$$

D_0_ = diameter (cm) of the FOA in the control.

D_x_ = diameter (cm) of the FOA in the test.

### 4.4. Genotoxicity Effect

To obtain blood samples, rats were anesthetized using pentobarbital, and samples were collected retro-orbitally using heparin-containing tubes. The collected blood (2 mL) from a male Wistar rat was mixed with an equal volume (2 mL) of Ca^II^- and Mg^II^-free phosphate-buffered saline (PBS) solutions (137 mM NaCl; 2.7 mM KCl; 10 mM Na_2_HPO_4_; 1.76 mM KH_2_PO_4_; pH 7.4). The diluted blood was then exposed to the samples. The samples were dissolved in PBS to achieve the desired concentrations (5 and 10 µg/mL). Subsequently, the diluted blood samples were incubated with the respective samples for two hours at 37 °C. The negative control was exposed to PBS, while hydrogen peroxide (250 µmol/L) was used as the positive control.

The comet assay, also known as the single-cell gel electrophoresis assay, is a commonly used technique to evaluate genotoxicity. It measures DNA damage in individual cells by visualizing the migration of fragmented DNA under an electric field. Several parameters are assessed in the comet assay, including tail moment, tail intensity, and tail length, which provide information about the extent and severity of DNA damage. Higher values of tail moment, tail intensity, and tail length indicate more significant genotoxic effects [57,58,59,60].

A modified version of the alkaline comet test protocol described in [61] was implemented with minor adjustments. Following treatment, the suspension was centrifuged at 4500 rpm for 10 min. The pellet containing leukocytes was then dissolved in PBS (1 mL) after removing the supernatant. The washing process was repeated three times. Subsequently, the pellet was dissolved in a solution of low-melting-point (LMP) agarose (0.5% *w*/*v* in PBS). The resulting mixture was applied to a slide previously coated with normal-melting-point (NMP) agarose (1.5% *w*/*v*). The slides were immersed in a lysis solution (2.5 M NaCl, 100 mM Na2EDTA, 20 mM Tris, 300 mM NaOH, 1% N-lauroylsarcosine sodium, 10% DMSO, and 1% Triton X-100) for 5 min, followed by 1 h of incubation in the dark at 4 °C. After the lysis period, the slides were thoroughly washed with double-distilled water. The slides were then placed in horizontal gel electrophoresis apparatus with an electrophoresis solution consisting of 300 mM NaOH and 1 mM Na_2_EDTA at pH 13. DNA unravelling was performed for 20 min at a constant current of 300 mA and a set voltage of 25 V. The temperature of the electrophoresis solution was maintained at 4 °C throughout the run. Following electrophoresis, the slides were immersed in a neutralization buffer (400 mM Trizma solution adjusted to pH 7.5 using HCl) for 5 min. This process was repeated three times. Finally, the comets were visualized using the ethidium bromide staining method [62].

The ethidium-bromide-stained slides were observed and captured using ZOE Cell Imager fluorescence microscopy, specifically utilizing the red channel with an excitation wavelength of 556/20 nm and an emission wavelength of 615/61 nm. To quantitatively estimate DNA damage, an image analyzer coupled with processing software was employed. In this study, Comet Assay IV image analysis software was utilized, enabling the quantification of various parameters associated with DNA lesions [63]. Two replicates were conducted for each sample, and a total of fifty cells were randomly selected for analysis in each replicate.

### 4.5. Statistical Analysis

Statistical analysis was performed using GraphPad Prism V9 software. A one-way ANOVA was utilized to assess the statistical significance of the data. Differences between treatment groups were examined using Tukey’s honest significance test, with significance thresholds set at *p* < 0.05, *p* < 0.01, *p* < 0.001, and *p* < 0.0001.

## 5. Conclusions

In this work, we have prepared two new molecules, 1,5-dimethyl-*N*-phenyl-1H-pyrazole-3-carboxamide (**L_1_**) and 1,5-dimethyl-*N*-propyl-1H-pyrazole-3-carboxamide (**L_2_**), based on pyrazoles and amides. Their reactivity with nickel and cadmium salts led to two complexes **C_1_** and **C_2_**. We further support our study with biological investigations that showcased our coordination complexes as potential antibacterial and antifungal agents. We demonstrated that **C_2_** has a remarkable antifungal activity compared to cycloheximide. This Cd coordination complex also exhibits a remarkable anti-Fusarium activity (96% inhibition) against *Fusarium Oxysporum* f. sp. *Albedinis*. We also investigated the genotoxicity of our ligands and coordination complexes using the comet assay, where we demonstrated that the addition of metal ions increased the genotoxicity, as expected, compared to our ligands. We believe that the development of less genotoxic coordination compounds with a high biological activity is an ideal future research direction.

## Figures and Tables

**Table 1:** Inhibition zones (mm) of bacterial targets obtained with **L_1,_ L_2_**, **C_1_**, **C_2_**, and other reference compounds.

Compound	C (mmol/L)	Inhibition Zone (mm)				Ref.
		*E. coli* ^[n]^	*Li. Innocua* ^[p]^	*Ps. Aeruginosa* ^[n]^	*S. Aureus* ^[p]^	
**L_1_**	27.8	9.20 ± 0.53 *	7.94 ± 0.40 *	9.17 ± 1.04 *	8.17 ± 0.76 *	This work
**C_1_**	7.98	9.65 ± 0.28 *	10.20 ± 0.28 *	10.15 ± 0.28 *	10.6 ± 0.28 *	This work
**L_2_**	33.1	9.30 ± 0.17 *	8.33 ± 1.15 *	9.17 ± 0.85 *	7.10 ± 1.56 *	This work
**C_2_**	7.88	16.10 ± 0.28 *	14 ± 0.28 *	14.7 ± 0.57 *	12.8 ± 0.28 *	This work
**Gentamicin**	1.25	29.9 ± 0.28	30.10 ± 0.28	27.75 ± 0.35	32.85 ± 0.70	This work
**[Cd_2_(dmamp)_2_ (NCS)_2_]_n_**	---	9.2	--	13.1	--	[37]
**[Ni(btbepa)CH_3_OH(H_2_O)](ClO_4_)_2_**	--	28	--	--	27	[6]
**H_3_O[Ni(dmptr)_3_](ClO_4_)_3_**	4.62	12 ± 0.38	--	--	12 ± 0.25	[32]
**[Cd(dmptr)_2_ Cl_2_]**	7.85	27 ± 0.25	--	--	27 ± 0.19	[32]
**[Cd(htcd)(SCN)]SCN**	--	22	--	--	24	[39]
**[NiL_3_](ClO_4_)_2_**	3.49	8 ± 0.8	9 ± 0.35	9 ± 0.95	8 ± 0.35	[38]
**CdL_2_Cl_2_**	5.25	14 ± 0.75	11 ± 0.5	12 ± 0.3	11 ± 0.5	[38]
**Ni(ClO_4_)_2_·6H_2_O**	8.2	0	0	0	0	[38]
**CdCl_2_·H_2_O**	14.9	11 ± 0.3	0	8 ± 0.2	0	[38]

^[p]^: Gram positive; ^[n]^: Gram negative. * *p* < 0.05 compared with Gentamicin. dmamp = 2-[(2-dimethylaminoethylimino)methyl]phenol). btbepa = *N*,*N*-bis(2-(10,5,50-trimethyl-1H,10H-[3,30-bipyrazol]-1-yl)ethyl)propan-1-amine. dmptr = 3-(3,5-dimethyl-1H-pyrazol-1-yl)-1H-1,2,4-triazole. htcd = 5,7,7,12,14,14-hexamethyl-1,4,8,11-tetraazacyclotetradeca-4,11-diene. L = 2-(3,5,5′-trimethyl-1′H-[1,3′-bipyrazol]-1′-yl)acetonitrile.

**Table 2:** Inhibition zones (mm) of fungal targets obtained with **L_1_**, **L_2_**, **C_1_**, and **C_2_** and comparison with the literature.

Compound	C (mmol/L)	Inhibition Zone (mm)			Ref.
		*G.candidum*	*A. niger*	*P. crustosum*	
**L_1_**	27.87	13.9 ± 0.1 *	10.25 ± 0.75 *	8.8 ± 0.2 *	This work
**C_1_**	7.98	15.5 ± 0.71 *	19 ± 1.41 *	22 ± 1.41	This work
**L_2_**	33.11	12.5 ± 0.6 *	9.85 ± 0.65 *	8.25 ± 0.25 *	This work
**C_2_**	7.88	20 ± 1.41 *	24.5 ± 0.71 *	26 ± 2.83 *	This work
**Cycloheximide**	21.33	22.8 ± 0.2	22.7 ± 0.5	22.6 ± 0.6	This work
**Cycloheximide (lower concentration)**	10.66	22 ± 0.8	22 ± 0.7	22 ± 0.6	This work
**[Ni(IND)_2_(Gly)(H_2_O) _2_]Cl**	--	22.7 ± 0.03	--	--	[41]
**Cd(bdade)(NCS)_2_**	--	--	18.44	--	[42]
**[Ni(psi)_2_SO_4_]**	--	--	10	--	[43]
**[Ni(CH_3_COO)_2_(Tyr)_2_] Tyr = Tyrosine**	--	--	23	--	[45]
**[NiL_3_](ClO_4_)_2_**	3.49	0	9	9 ± 0.1	[38]
**CdL_2_Cl_2_**	5.25	14 ± 0.2	14 ± 0.8	15 ± 0.45	[38]
**Ni(ClO_4_)_2_·6H_2_O**	8.2	0	0	0	[38]
**CdCl_2_·H_2_O**	14.9	10 ± 0.1	6 ± 0.3	5 ± 0.2	[38]

* *p* < 0.05 compared with cycloheximide (T+). IND = indapamide; Gly = glycine; bdade = bis(3(4-dimethylaminophenyl)-allylidene)-1,2-diaminoethane; psi = hydrazine carboxamide, 2-[3-methyl-2-thienylmethylene]; L = 2-(3,5,5′-trimethyl-1′H-[1,3′-bipyrazol]-1′-yl) acetonitrile.

**Table 3:** Antifusariam activities of **L_1_**, **L_2_**, **C_1_**, **C_2_**, and reference compounds for different sample volumes.

Compound	Sample Volumes (µL)	C (µmol/L)	Inhibition (%)	Ref.
**L_1_**	50	92.9	46.3 ± 2.5 *	This work
	150	278.7	50 ± 4.5	
**C_1_**	50	26.6	43.8 ± 3.3	This work
	150	79.8	62.5 ± 2.2 *	
**L_2_**	50	110.35	50 ± 7.8 *	This work
	150	331.05	57.5 ± 6.6	
**C_2_**	50	26.3	87.5 ± 3.3 *	This work
	150	78.9	96.3 ± 1.3 *	
**Cycloheximide**	50	71.1	35 ± 3.3	This work
	150	213.3	55 ± 2.5	
	250	355.5	66.7 ± 2.9	
**Cd(** **dmptr** **)_2_ Cl_2_**	200	104.6	50	[32]
**(H_3_O)[Ni(** **dmptr** **)_3_](ClO_4_)_3_**	200	29.41	38	[32]
**[Ni(** **btbepa** **)CH_3_OH(H_2_O)](ClO_4_)_2_**	200	68.8	44	[6]
**NiL_3_(ClO_4_)_2_**	150	66.42	35	[38]
**CdL_2_Cl_2_**	150	97.75	51	[38]
**Co_2_(HL_2_)L_2_Cl_3_**	150	81.1	97	[38]
**Cu_2_(HL’_2_)Cl_2_**	150	158.02	97	[44]
**Ni(ClO_4_)_2_·6H_2_O**	150	164.07	8	[38]
**CdCl_2_·H_2_O**	150	298.01	12	[38]

* *p* < 0.05 compared to cycloheximide (T+), btbepa = *N*,*N*-bis(2-(10,5,50-trimethyl-1H,10 H-[3,30-bipyrazol]-1-yl)ethyl) propan-1-amine; dmptr = 3-(3,5-dimethyl-1H-pyrazol-1-yl)-1H-1,2,4-triazole; L = 2-(3,5,5′-trimethyl-1′H-[1,3′-bipyrazol]-1′-yl)acetonitrile; HL2 = 1′-((1H-tetrazole-5-yl)methyl)-3,5,5′-trimethyl-1′H-1,3′-bipyrazole; HL2′ = 5-((3-(3,5-dimethyl-1H-pyrazol-1-yl)-1H-1,2,4-triazol-1-yl)methyl)-406 1H-tetrazole.

**Table 4:** Crystal data and structure refinement for **L_1_**, **C_1_**, and **C_2_**.

Compound	L_1_	C_1_	C_2_
**Empirical formula**	C_12_ H_13_ N_3_ O	C_26_ H_34_ Cl_2_ N_6_ Ni O_12_	C_38_ H_68_ Cd_4_ Cl_8_ N_12_ O_6_
**Formula weight**	215.25	752.20	1522.24
**T (K)**	293(2)	293(2)	293(2)
**Wavelength (Å)**	0.71073	0.71073	0.71073
**Crystal system**	Orthorhombic	Monoclinic	Orthorhombic
**Space group**	*Pbca*	*P*2_1_/*n*	*Pca*2_1_
**Unit cell dimensions (Å,°)**	a = 10.4982(4)	a = 10.6244(6)	a = 16.7846(3)
	b = 9.8653(4)	b = 13.0110(7)	b = 16.4470(3)
	c = 21.4838(9)	c = 11.8231(7)	c = 20.9709(3)
	α = 90	α = 90	α = 90
	β = 90	β = 96.503(6)	β = 90
	γ = 90	γ = 90	γ = 90
**V (Å^3^)**	2225.04(15)	1623.84(16)	5789.16(16)
**Z**	8	2	4
**Density (calculated) (g/cm^3^)**	1.285	1.538	1.747
**Absorption coefficient (mm^−1^)**	0.085	0.832	1.869
**F(000)**	912	780	3024
**Crystal size (mm^3^)**	0.32 × 0.15 × 0.12	0.35 × 0.30 × 0.20	0.40 × 0.25 × 0.20
θ**range for data collection (°)**	2.988 to 26.193	2.902 to 26.232	2.893 to 26.187
**Reflections collected**	16410	11040	39165
**Independent reflections**	2215 [R(int) = 0.0480]	3199 [R(int) = 0.0253]	11,407 [R(int) = 0.0185]
**Completeness to** θ **25.242° (%)**	99.2	98.9	98.9
Max. and min. transmission	1.00000 and 0.82569	1.00000 and 0.88273	1.00000 and 0.66350
Data/restraints/parameters	2215/0/147	3199/99/273	11407/43/721
Goodness-of-fit on F^2^	1.086	1.045	1.070
Final R indices [I > 2s(I)]	R1 = 0.0441, wR2 = 0.1216	R1 = 0.0374, wR2 = 0.1059	R1 = 0.0188, wR2 = 0.0486
R indices (all data)	R1 = 0.0488, wR2 = 0.1254	R1 = 0.0398, wR2 = 0.1079	R1 = 0.0197, wR2 = 0.0490
Absolute structure parameter	.	.	0.31(2)
Δρ(max, min)(e.Å^−3^)	0.187, −0.170	0.411, −0.289	0.528, −0.371

## Data Availability

Data are contained within the article and Supplementary Materials.

## Supplementary Materials

The following supporting information can be downloaded at: https://www.mdpi.com/article/10.3390/molecules29051186/s1, Figures S1–S4: NMR spectroscopy of **L_1_**, **L_2_**, **C_1_**, **C_2_**. Figures S5–S8: mass spectroscopy of **L_1_**, **L_2_**, **C_1_**, **C_2_.**
