# Supplementary material for: Synthesis, Crystal Structures, Genotoxicity, and Antifungal and Antibacterial Studies of Ni(II) and Cd(II) Pyrazole Amide Coordination Complexes
Source: Molecules. 2024 Mar 6;29(5):1186. doi: 10.3390/molecules29051186 (PMC10935173; doi:10.3390/molecules29051186)
Supplement: Supplementary file 1 [file molecules-29-01186-s001.zip › molecules-2879992-supplementary.pdf]

Electronic Supplementary Information (ESI)

Synthesis, Crystal Structures, Genotoxicity, and Antifungal and Antibacterial Studies of Ni(II) and Cd(II) Pyrazole Amide Coordination Complexes

Amal El Mahdaoui,[1] Smaail Radi,[1*] Youssef Draoui,[1] Mohamed El Massaoudi,[1] Sabir Ouahhoud,[2,3] Abdeslam Asehraou,[2] Nour eddine Bentouhami,[2], Ennouamane Saalaoui,[2] Redouane Benabbes,[2] Koen Robeyns,[4] Yann Garcia,[4*]

[1] LCAE, Department of Chemistry, Faculty of Sciences, University Mohammed I, Oujda 60 000, Morocco. E-mail: s.radi@ump.ac.ma

[2] Laboratory of Bioresource Biotechnology Ethnopharmacology and Health Faculty of Sciences, University Mohammed I, Oujda 60 000, Morocco. a.asehraou@ump.ac.ma

[3] Faculty of Medicine and Pharmacy, University Sultan Moulay Slimane, Beni Mellal, 23000, Morocco.

[4] Institute of Condensed Matter and Nanosciences, Molecular Chemistry, Materials and Catalysis (IMCN/MOST), Université catholique de Louvain, Place Louis Pasteur 1, 1348 Louvain-la-Neuve, Belgium. E-mail: yann.garcia@uclouvain.be

Contents:

- **Figure S1.** ^{1}H NMR spectrum of **L_1**
- **Figure S2.** ^{13}C NMR spectrum of **L_1**
- **Figure S3.** ^{1}H NMR spectrum of **L_2**
- **Figure S4.** ^{13}C NMR spectrum of **L_2**
- **Figure S5.** Mass spectroscopy analysis of **L_1**
- **Figure S6.** Mass spectroscopy analysis of **L_2**
- **Figure S7.** Mass spectroscopy analysis of **C_1**
- **Figure S8.** Mass spectroscopy analysis of **C_2**

Figure S1. ^{1}H NMR spectrum of $\mathbf{L_1}$

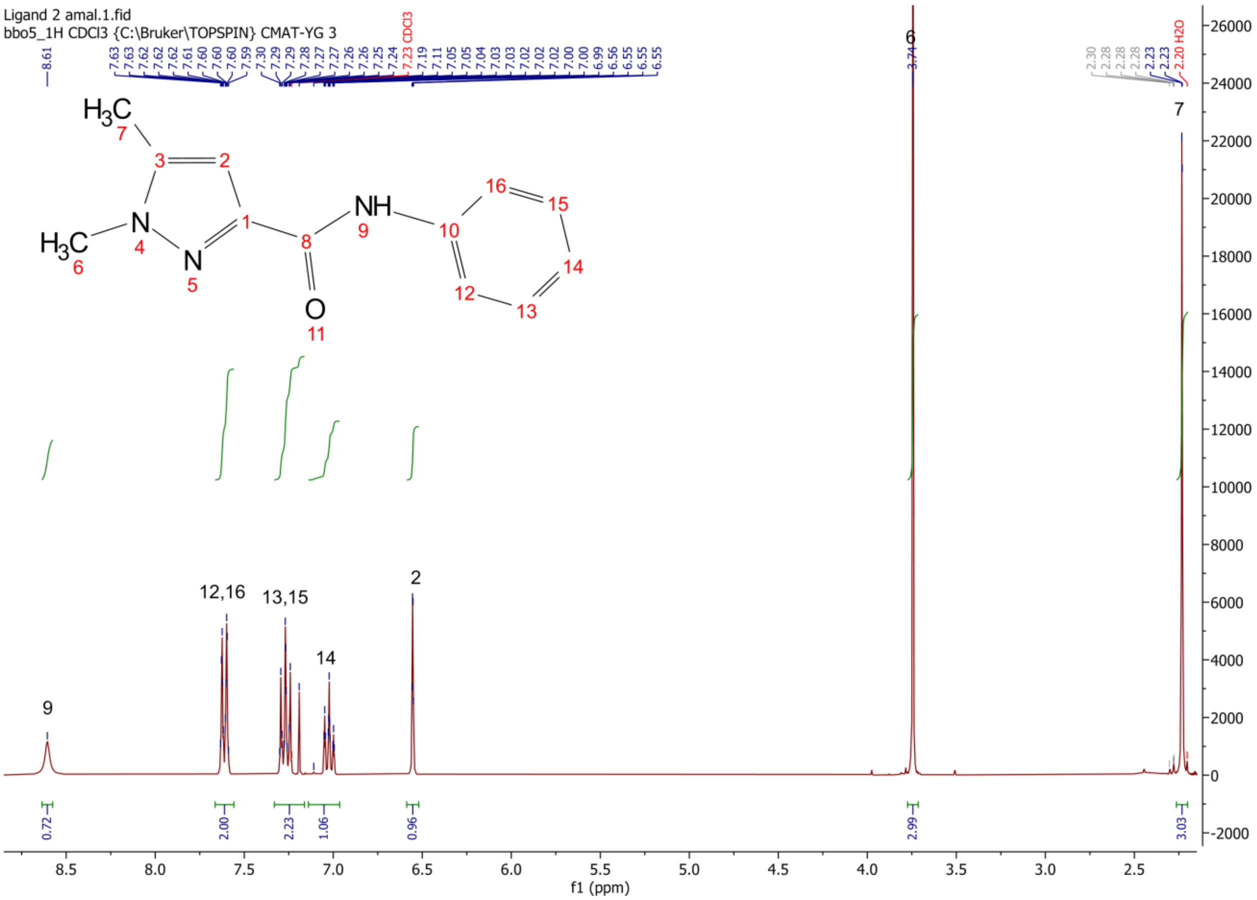

Figure S2. ^{13}C NMR spectrum of **L_1**

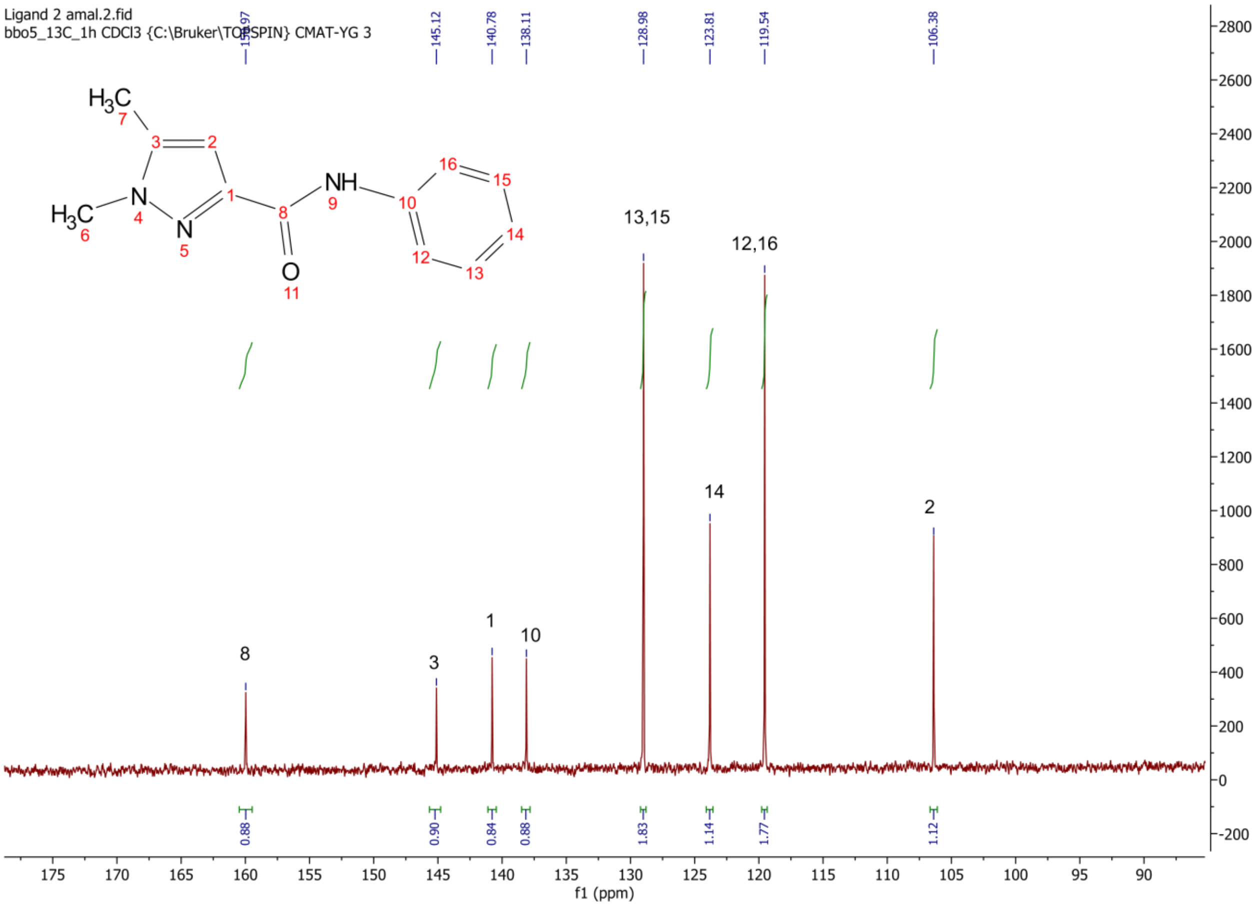

Figure S3. ^{1}H NMR spectrum of **L$_2$**

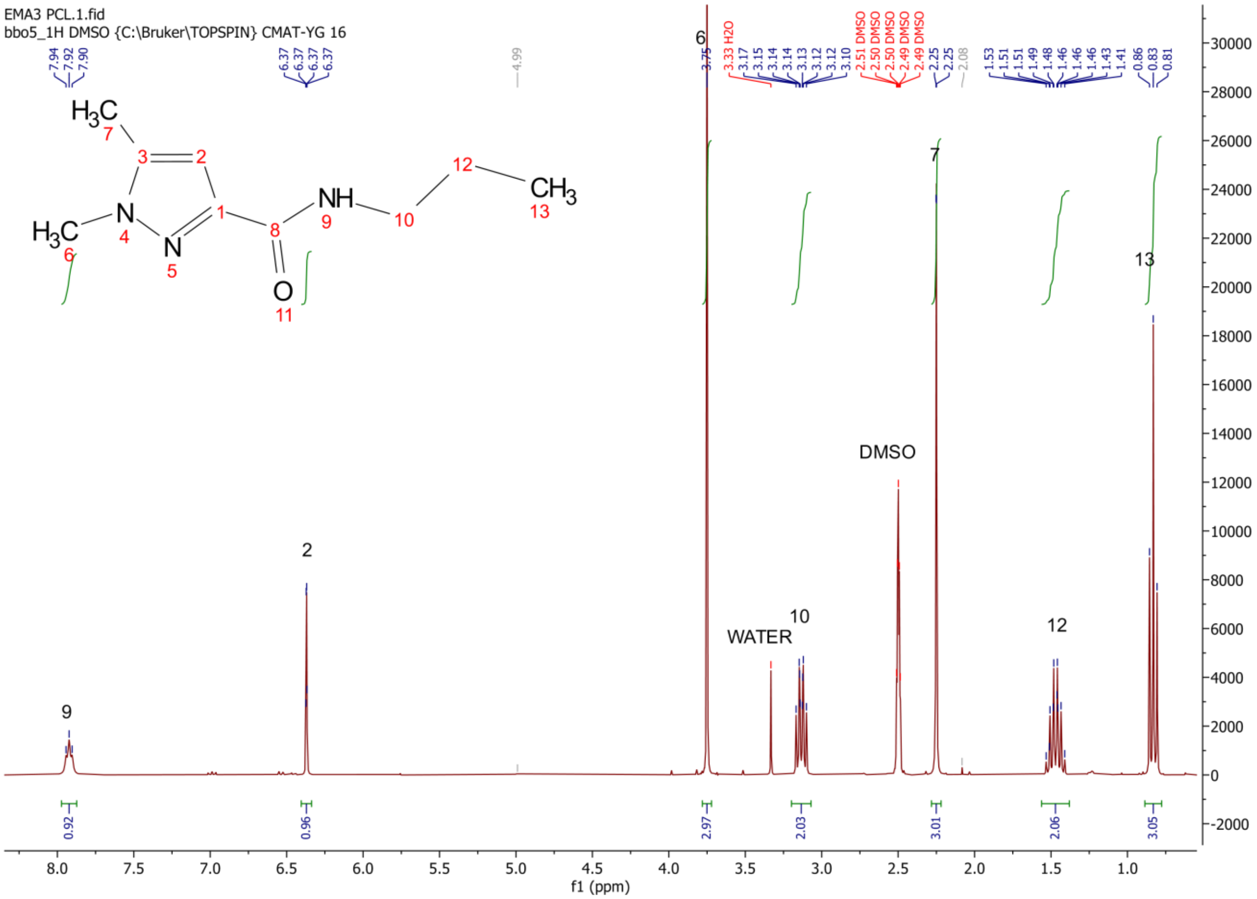

Figure S4. ^{13}C NMR spectrum of **L_2**

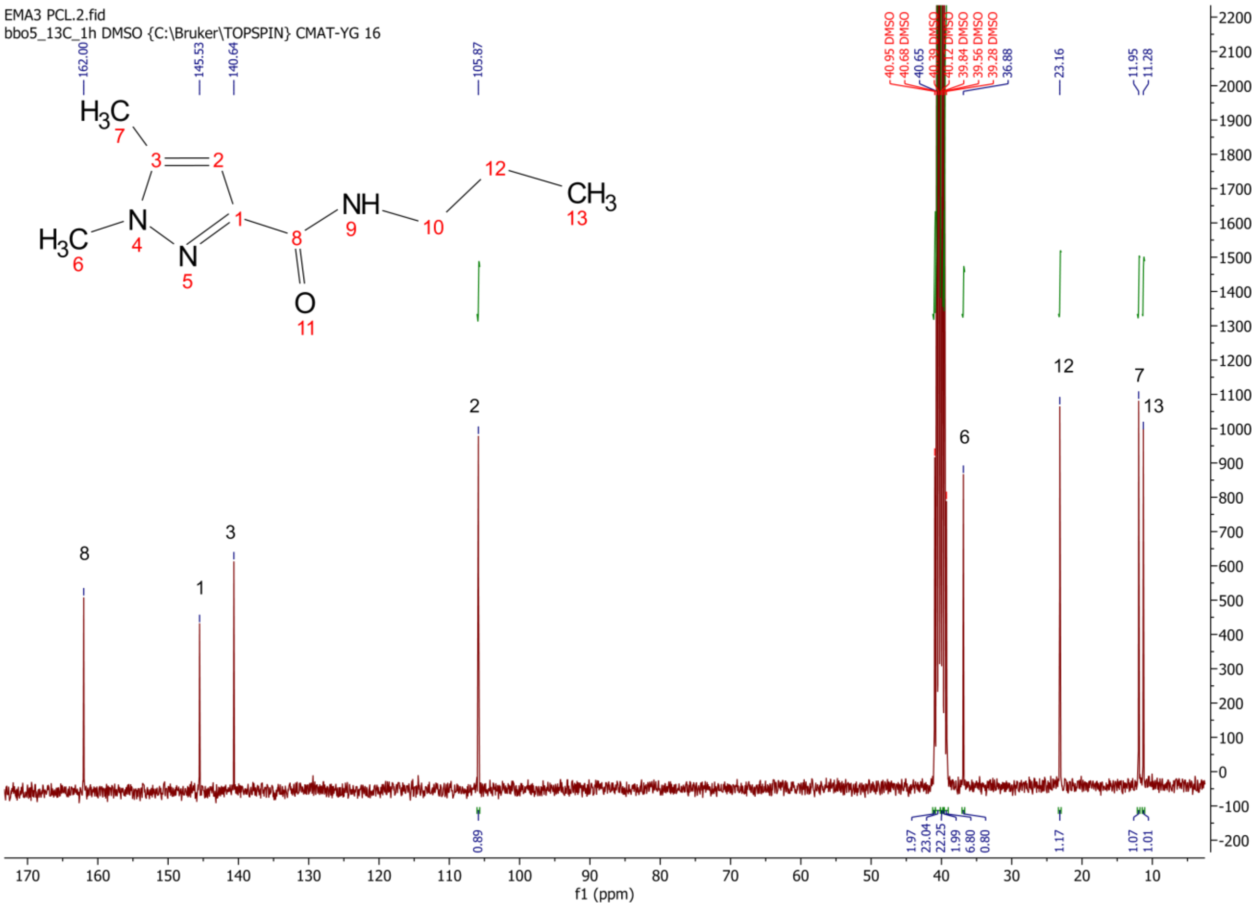

Figure S5. Mass spectroscopy analysis of $\mathbf{L_1}$

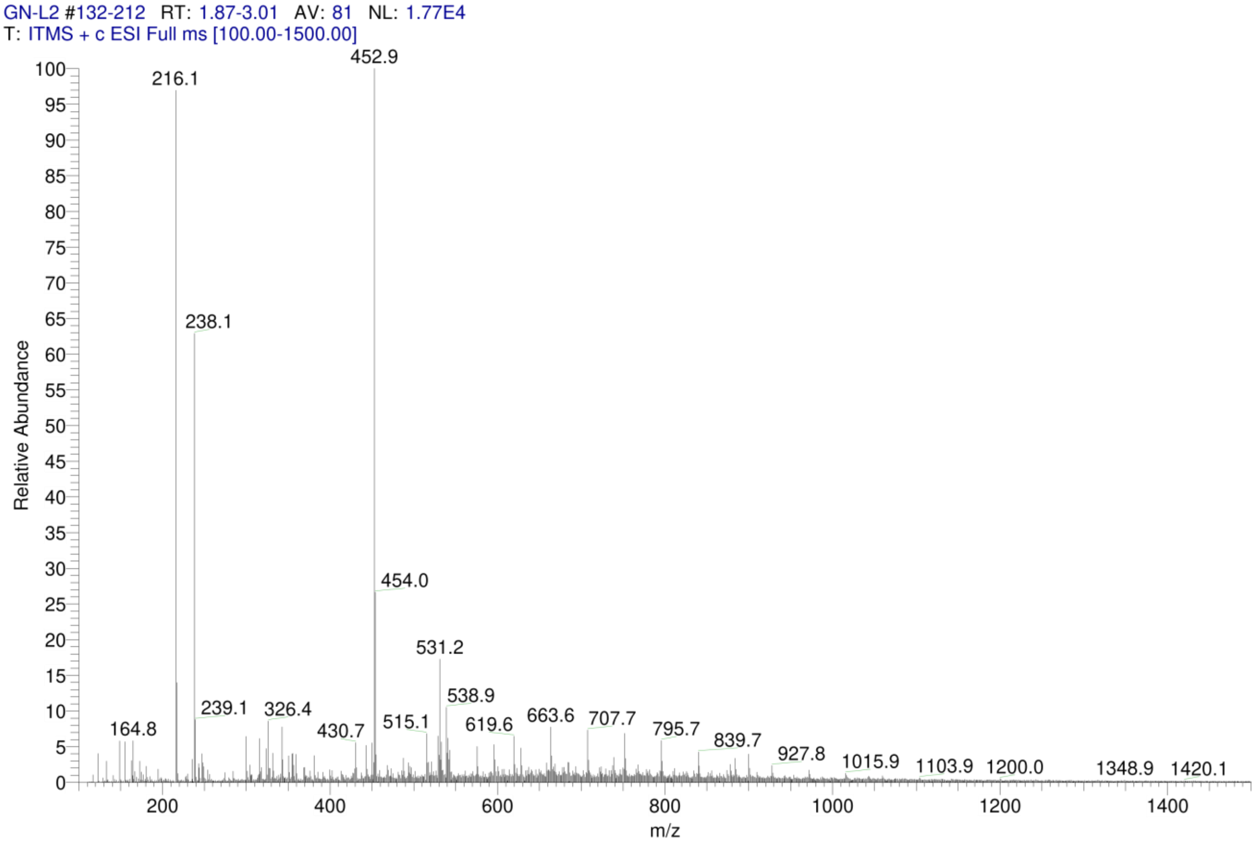

Figure S6. Mass spectroscopy analysis of **L_2**

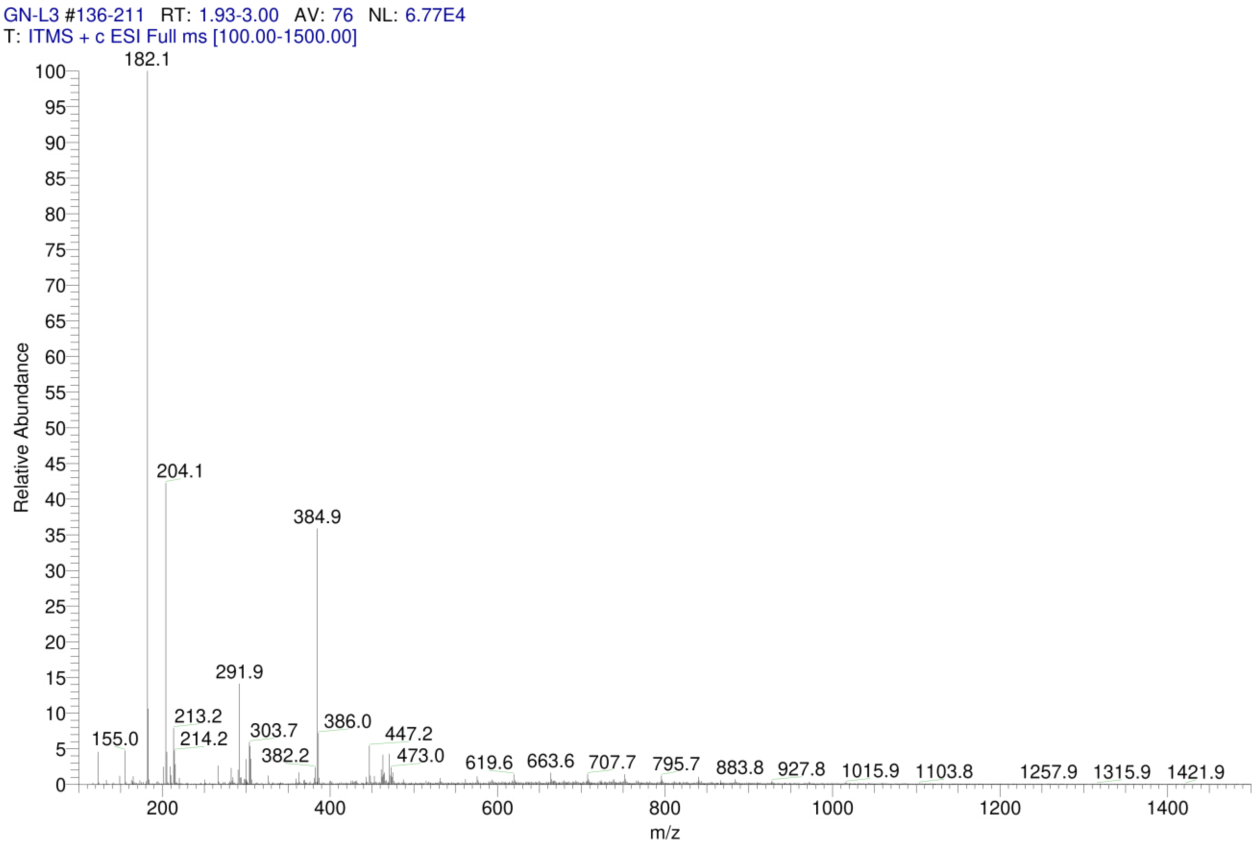

Figure S7. Mass spectroscopy analysis of **C_1**

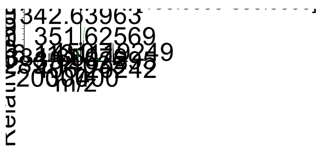

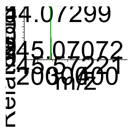

Peak Mass	Display Formula	MS Cov. [%]	Delta [ppm]	Theo. mass
244.07280	$C_{24}H_{26}O_2N_6{}^{58}Ni$	72.20	-0.78	244.07299

Figure S8. Mass spectroscopy analysis of **C_2**

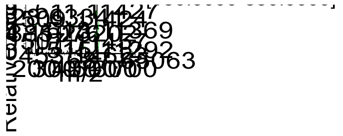

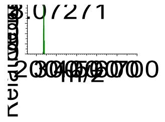

Peak Mass	Display Formula	MS Cov. [%]	Delta [ppm]	Theo. mass
234.07423	$C_{18}H_{30}O_2N_6{}^{106}Cd$	99.24	0.16	234.07419
